# NSD2 inhibits the expression of PD-L1 via oxidative phosphorylation to control immune surveillance in hepatocellular carcinoma

**DOI:** 10.1038/s41419-026-08490-x

**Published:** 2026-02-27

**Authors:** Wei Zhang, Wenxin Feng, Chunxiao Ma, Hanyu Rao, Changwei Liu, Yue Xu, Ningyuan Liu, Ziyi Wang, Rebiguli Aji, Ting Han, Wei-Qiang Gao, Xiuying Xiao, Li Li

**Affiliations:** 1 State Key Laboratory of Systems Medicine for Cancer, Ren Ji Hospital, Shanghai Cancer Institute, School of Medicine and School of Biomedical Engineering, Shanghai Jiao Tong University, Shanghai, China; 2 School of Biomedical Engineering and Med-X Research Institute, Shanghai Jiao Tong University, Shanghai, China; 3 Department of Oncology, Ren Ji Hospital, School of Medicine, Shanghai Jiao Tong University, Shanghai, China

**Keywords:** Cancer genetics, Cell biology

## Abstract

Hepatocellular carcinoma (HCC) is the fourth most common cause of cancer-related death, and patients usually exhibit impaired immune function within the tumor environment. NSD2 is an H3K36 methyltransferase and has been considered a cancer-promoting factor. However, the role of NSD2 in the occurrence and development of HCC is still unclear. In this study, the effects of NSD2 on HCC were assessed by both mouse and cell models. RNA-seq, ChIP-seq, and orthotopic tumor models were employed to decipher the downstream mechanisms of NSD2 responsible for HCC development. NSD2 alterations were characterized in patients with HCC. Hepatocyte-specific NSD2 overexpression suppresses the proliferation of tumor cells in DEN-treated mice. Mechanistically, NSD2 inhibits OXPHOS by activating target genes (Camk2d and Prkce) transcription. Downregulation of OXPHOS, caused by overexpression of NSD2, inhibits the expression of PD-L1 and enhances immune recognition of tumors. What’s more, inhibition of OXPHOS suppresses the formation of HCC. Finally, patients with low expression of NSD2 have a better response to PD-L1 inhibitor treatment. These findings showed that NSD2 inhibits the progression of HCC by inhibiting the expression of PD-L1 through OXPHOS. Our results identify NSD2 as a tumor suppressor in the development of HCC.

## Introduction

HCC accounts for 85–90% of primary liver cancer [1]. In the past few decades, considerable progress has been made in the epidemiology, pathogenic factors, and molecular mapping of HCC [2]. At present, morbidity and cancer-specific mortality continue to increase in many countries [3]. In many parts of the world, most HCC patients are diagnosed at an advanced stage, so the 5-year survival rate of HCC is low [4]. Therefore, the underlying molecular mechanisms of HCC need to be further studied.

Current studies have demonstrated that the silencing of tumor suppressor genes mediated by promoter DNA hypermethylation and histone deacetylation plays a crucial role in the initiation and progression of hepatocellular carcinoma (HCC) [5, 6]. Specifically, aberrant hypermethylation of CpG islands within tumor suppressor gene promoters directly impedes transcriptional activation, while histone deacetylation condenses chromatin structure to further repress gene expression—both events synergistically disrupt normal cellular homeostasis and drive hepatocarcinogenesis [7]. NSD2 (nuclear receptor binding SET domain-containing protein 2) is known as WHSC1 (Wolf–Hirschhom syndrome candidate 1) or MMSET (multiple myeloma SET protein) [8]. NSD2 is a member of the histone lysine methyltransferase family [9]. Abnormal methylation of H3K36 mediated by NSD2 can lead to epigenetic regulation disorder [10, 11]. Recent studies have found that NSD2 is highly expressed in a variety of cancers, such as glioma, neuroblastoma, endometrial tumor, prostate cancer, gastric cancer, colorectal cancer, bladder cancer, and skin cancer [12, 13]. All these argue for NSD2 as an important player in the development of cancer. However, the role of NSD2 in the progression of HCC is still unclear.

Mounting research findings reveal that histones regulate OXPHOS via interactions with specific regulatory proteins and corresponding modification enzymes, and this regulatory mechanism is closely linked to the development of diseases such as cancer and chemoresistance [14]. For example, in prostate cancer, the histone methyltransferase ASH1L forms a complex with HIF-1α to induce the secretion of IGF-2 by mediating H3K4 and H3K36 methylation; IGF-2 then induces the differentiation of macrophages into lipid-associated tumor-associated macrophages (LA-TAMs) and promotes OXPHOS metabolic reprogramming in these cells to facilitate bone metastasis [15]. Mitochondrial oxidative phosphorylation (OXPHOS), as one of the energy supply pathways, plays an irreplaceable role in the development of tumor cells. Over the past century, glycolysis was once considered to be the main metabolic pathway that all tumor cells rely on to meet energy needs. However, in recent years, more and more studies have shown that some tumor subtypes mainly rely on OXPHOS rather than glycolysis to achieve proliferation and survival [16, 17]. In acute myeloid leukemia cells, inhibition of OXPHOS effectively induced myeloid differentiation and reduced stemness potential [18, 19]. However, the mechanism of OXPHOS in the tumorigenesis of HCC remains unclear.

High expression of programmed death ligand-1 (PD-L1), an immune checkpoint molecule, is an important feature for tumor cells to evade immune surveillance [20]. Accumulating research has revealed that PD-L1 is subject to regulation by the OXPHOS. It has been reported that inhibition of OXPHOS improves the efficacy of combined immunotherapy in renal cell carcinoma [21]. Moreover, OXPHOS can promote the expression of PD-L1 in macrophages to suppress inflammation, which revealed the regulatory effect of OXPHOS on PD-L1 [22]. These studies show that OXPHOS plays an important role in immune regulation.

In this study, we found that NSD2 inhibits the occurrence and development of HCC. In short, NSD2 inhibits the expression of PD-L1 in HCC cells by suppressing OXPHOS, so that tumor cells can be better recognized and killed by immune cells. Our data explain the mechanism by which NSD2 regulates the immune surveillance in HCC, which provides a novel strategy for the development of HCC-targeted drugs.

## Materials and methods

### Mice

All mice were fed at a specific pathogen-free facility. NSD2 OE/+ mice were gifted by Prof. Jun Qin (Chinese Academy of Sciences, Shanghai, China) [23]. Alb-Cre mice were purchased from Shanghai Model Organism. NSD2LVOE/+ mice were generated by crossing NSD2 OE/+ mice with Alb-Cre mice. Mice were kept on a C57BL/6 J background. Mouse experimental protocols were approved by the Renji Hospital Animal Care and Use Committee (202201027).

Nude mice (4–6 weeks old) were subcutaneously injected with 1 × 10⁶ hepa 1–6 cells (resuspended in 20 μL PBS/Matrigel, 1:1) into the right flank. Mice were euthanized when tumors reached 15 mm in maximum diameter. Tumor tissues were harvested, weighed, and processed for subsequent analyses. To establish the diethylnitrosamine (DEN)-induced hepatocellular carcinoma (HCC) model, 2-week-old male C57BL/6 mice were intraperitoneally (i.p.) injected with a single dose of DEN at 25 mg/kg body weight. Following the injection, the mice were housed in a specific pathogen-free (SPF) facility under standardized conditions (22 ± 2°C, 50 ± 10% humidity, 12-h light/dark cycle) with free access to sterile water and standard chow for the predetermined experimental duration. For the orthotopic hepatocellular carcinoma (HCC) model, 6–8-week-old male C57BL/6 mice (SPF conditions) were anesthetized, a midline abdominal incision was made to expose the liver, and 1 × 10⁶ logarithmic-phase HCC cells (resuspended in 20 μL PBS/Matrigel mixture) were injected into the left lateral lobe using a 30-gauge syringe, followed by pressure application to prevent leakage and layered suture closure. For oligomycin treatment, drugs were dissolved in 5% DMSO (Sigma-Aldrich) and 95% corn oil (Sigma-Aldrich) and injected intraperitoneally at a dose of 0.1 mg/kg for oligomycin (Macklin Cat#O815255) once daily and euthanized at day 10. For PD-L1 blockade treatment, the mice were treated with PBS (CON), and anti-mouse PD-L1 (BioXCell Cat# BE0101, 5 mg/kg per mouse) every other 3 days and euthanized at day 10. The antibodies used in the experiment are shown in the Supplementary Materials (Supplementary Table 2).

### Cell lines

293 T, Hepa 1–6, and HepG2 cell lines were cultured in DMEM (Gibco). Media were added with 1% penicillin–streptomycin solution (HyClone) and 10% fetal bovine serum (FBS; Biological Industries). All cell lines were cultured in an incubator at 37 °C with 5% CO_2_.

Glucose consumption (Biovision, Cat#K686-100), lactate accumulation (Biovision, Cat#K627-100), glycolysis (Abcam, Cat#ab197244), and Oxygen consumption (abcam, Cat#ab197243) were detected by a commercial kit.

### Reverse transcription and qPCR

For tissue RNA extraction, 30 mg of tissue was cut and harvested by centrifugation. Total RNA was extracted with an RNA extraction kit (Bio Dete). Totally, 1 μg of extracted RNA was used for reverse transcription. The cDNA was then assayed by qPCR. Gapdh was used for qPCR normalization. The primers used in the experiment are shown in the Supplementary Materials (Supplementary Table 1). Assays were repeated at least three times. *P* value was calculated using the Student *t*-test.

### ChIP-seq analysis

Filtering process was conducted using Cutadapt v1.18 with non-default parameters (--max-n 0 --minimum-length 35) and Trimmomatic v0.38 with non-default parameters (SLIDINGWINDOW:4:15 LEADING:10 TRAILING:10 MINLEN:35). Subsequently, FastQC was run with default parameters to validate the high quality of the resulting reads. The clean reads were then aligned to the mouse genome (assembly GRCm38) using Bowtie2 v2.3.4.1 under default parameter settings, followed by the removal of duplicate reads via Picard MarkDuplicates. Peak detection was carried out using the MACS v2.1.2 peak-calling algorithm with non-default parameters (-f BAMPE -g hs/mm -p 0.01), where a *p*-value cutoff of 0.01 was applied. Finally, annotation of peak sites to corresponding gene features was performed using the ChIPseeker R package.

### Statistical analysis

Student’s *t*-test was used to analyze the experimental data, and the results were expressed as mean ± s.e.m (SEM). For oxygen consumption and glycolysis analysis, differences between groups were analyzed by two-way ANOVA. For qRT-PCR analysis of a panel of genes, data are presented as mean ± standard deviation (SD) from three independent biological replicates. Statistical significance was assessed using two-tailed unpaired multiple *t*-tests, with false discovery rate (FDR) correction applied to control for Type I errors. Corresponding q-values are indicated above each pair of bars. A *q*-value < 0.05 was considered statistically significant. The relationship between NSD2 and gene expression was evaluated by the Pearson correlation coefficient. In the survival analysis, Cox proportional hazards regression analysis was employed to adjust for confounding factors. GraphPad Prism software was used for statistical analysis. All data sets with *p*-values less than 0.05 were considered statistically significant.

## Results

### NSD2 suppresses the formation of HCC in mice

**Fig. 1:**
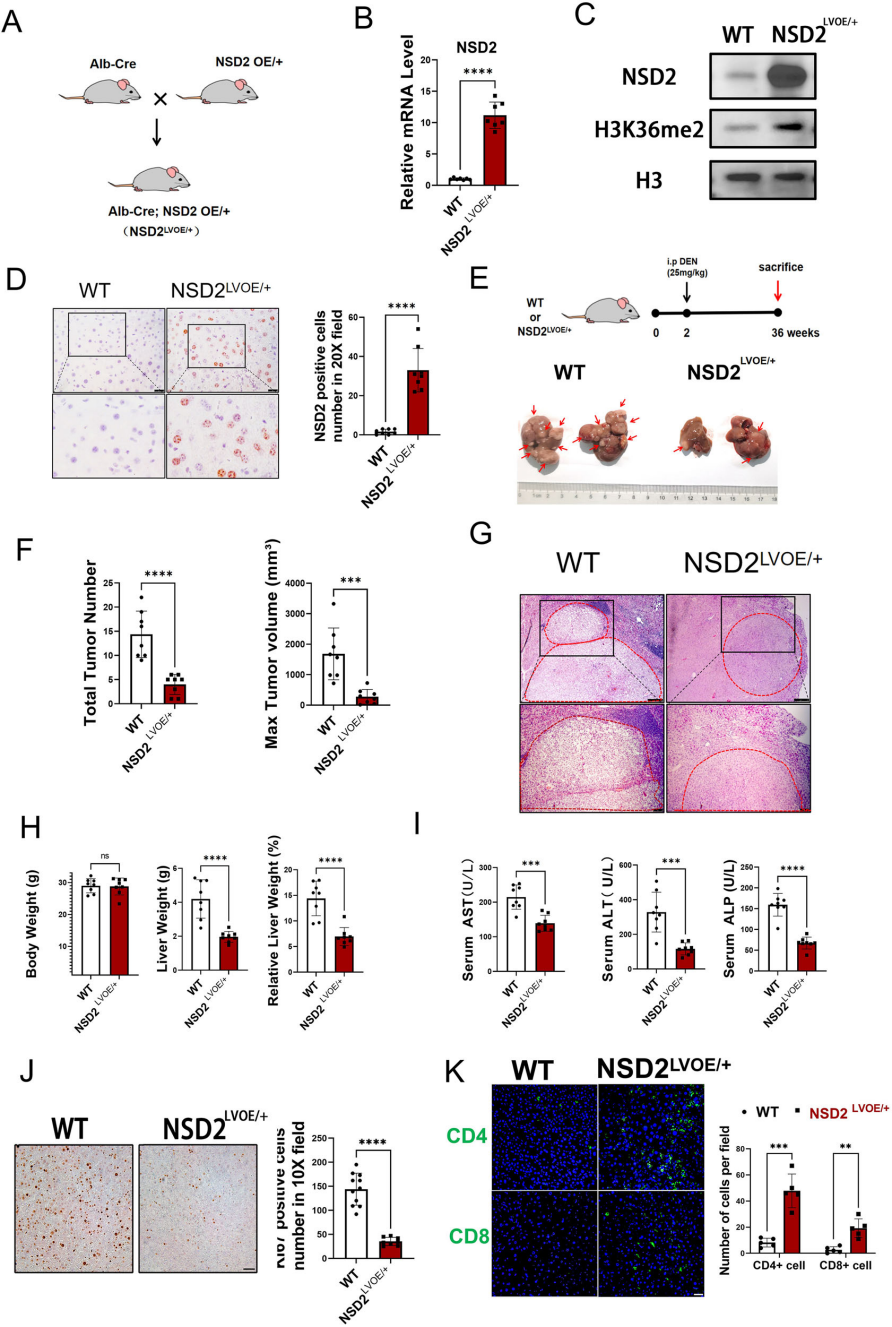
NSD2 suppresses the formation of HCC in mice. **A** Schematic representation of the generation of hepatocyte-specific NSD2 overexpression mouse strain. **B** Relative mRNA expression levels of NSD2 in liver from WT and NSD2LVOE/+ mice (*n* = 8 per genotype). **C** Immunoblot analyses of NSD2 and H3K36me2 expression in liver from WT and NSD2LVOE/+ mice are shown (repeated more than three times). **D** Immunohistochemical analyses of NSD2 expression are shown (scale bars: 75 μm). **E** Representative images of liver from WT and NSD2LVOE/+ mice. **F** Tumor number and max tumor volume of liver from WT and NSD2LVOE/+ mice (*n* = 8, per group). **G** Representative HE staining of liver tissue from 9-month-old WT and NSD2LVOE/+ mice with DEN treatment (the area within the red circle indicates the tumor tissue. Scale bars: 75 μm). **H** Body weights, liver weights and relative liver weights of 9-month-old WT and NSD2LVOE/+ mice with DEN treatment (*n* = 8, per group). **I** Quantification of serum AST, ALT, and ALP of 9-month-old mice with DEN treatment (*n* = 8, per group). **J** Representative Ki67 staining and quantitation of Ki67 positive cells in liver tissue from 9-month-old WT and NSD2LVOE/+ mice with DEN treatment (scale bars: 75 μm). **K** Representative CD4 and CD8 staining and quantitation of positive cells in liver tissue from 9-month-old WT and NSD2LVOE/+ mice with DEN treatment (scale bars: 75 μm).

It has been reported that NSD2 can promote the proliferation of HCC cell lines in vitro [24]. To confirm this, we first constructed a Hepa 1–6 cell line with NSD2 overexpression and conducted a subcutaneous experiment in nude mice. The results showed that NSD2 overexpression promoted the growth of Hepa 1–6 in nude mice (Supplementary Fig. 1A, B).To further evaluate the role of NSD2 in HCC, we crossed NSD2 OE/+ mice^19^ with Alb-Cre mice to obtain a hepatocyte-specific NSD2 overexpression mouse strain (Alb-Cre; NSD2 OE/+ mice, hereinafter referred to as NSD2^LVOE/+^ mice) (Fig. 1A). NSD2 OE/+ (hereinafter referred to as WT) mice were used as the control in the following experiments. As expected, the expression of NSD2 was efficiently upregulated, and H3K36me2 was substantially increased in the liver of NSD2^LVOE/+^ mice by immunoblot and immunohistochemical staining validation (Fig. 1B–D). No visible tumors or liver weight change were observed in the livers of 9-month-old mice, either control or NSD2^LVOE/+^ mice (Supplementary Fig. 1C, D). Next, we injected DEN i.p. into WT and NSD2^LVOE/+^ mice at age 2 weeks, which were euthanized at 36 weeks (Fig. 1E). Surprisingly, WT mice developed many large liver tumor nodules from 36 weeks, which is inconsistent with the previous results in nude mice. Meanwhile, only a few small liver tumors appeared in NSD2^LVOE/+^ mice (Fig. 1E–G). We also observed lighter liver weight in NSD2^LVOE/+^ mice than in the control group (Fig. 1H). Compared with control mice, the HCC mark Afp, and molecular marks for liver damage, AST and ALT were much lower in the serum of NSD2^LVOE/+^ mice (Fig. 1I). In order to further evaluate the severity of tumorigenesis in NSD2^LVOE/+^ mice, the proliferation of hepatocytes was detected by immunohistochemical staining. The results showed that the number of Ki67-positive cells in the liver tissue of NSD2^LVOE/+^ mice was significantly decreased compared with control mice (Fig. 1J). Considering that liver fibrosis is an important precancerous lesion in the development of HCC, we then performed Masson staining. The results showed that the fibrosis area in the liver tissue of NSD2^LVOE/+^ mice was significantly less than that in control mice (Supplementary Fig. 1E). Finally, Immunofluorescence revealed that there is more infiltration of NK cells, CD4+ and CD8+ T cells in HCC tissues of NSD2LVOE/+ mice(Fig. 1K and Supplement Fig. 1F). There is no significant change in macrophage infiltration in HCC tissues of NSD2LVOE/+ mice (Supplement Fig. 1F). These findings demonstrated that NSD2 suppresses the formation of HCC in immunocompetent mice.

### NSD2 inhibits OXPHOS and decreases glucose consumption in HCC cells

**Fig. 2:**
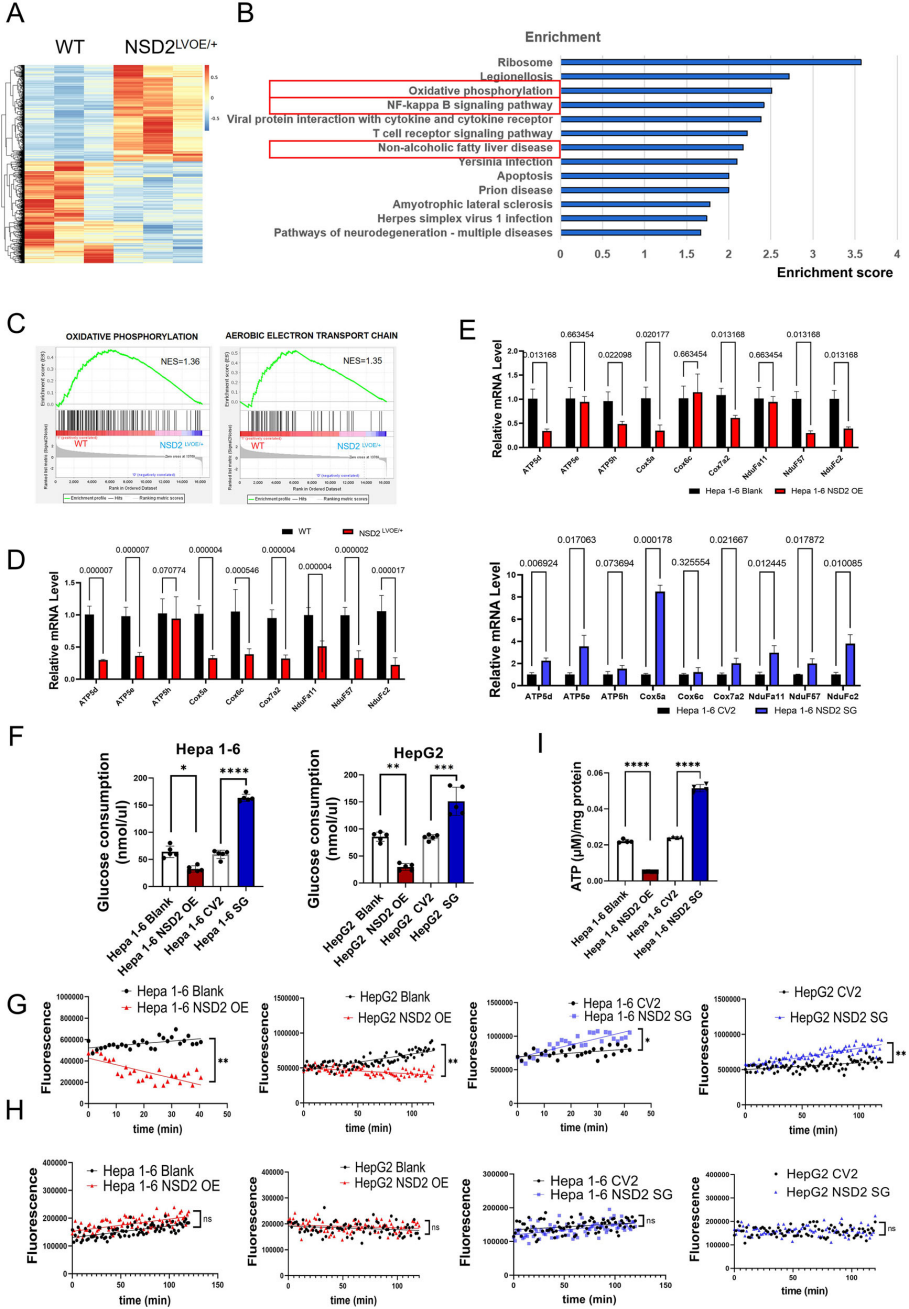
NSD2 inhibits OXPHOS and decreases glucose consumption in HCC cells. **A** Heat map of RNA-seq data to compare the gene expression in the liver from WT and NSD2LVOE/+ mice of 2-week-old mice with DEN treatment (*n* = 3). **B** KEGG term analysis of gene expression changes in the liver from WT and NSD2LVOE/+ mice of 2-week-old mice with DEN treatment. **C** GSEA plot of enrichment in WT and NSD2LVOE/+ liver. **D** Relative mRNA expression levels of OXPHOS in liver from DEN-treated[2w] mice as indicated (*n* = 6 per genotype). Data are presented as mean ± standard deviation (SD) from three independent biological replicates. Statistical significance was assessed using two-tailed unpaired multiple *t*-tests, with false discovery rate (FDR) correction applied to control for Type I errors. Corresponding *q*-values are indicated above each pair of bars. *q* < 0.05 was considered statistically significant. **E** NSD2 was knocked out or overexpressed in Hepa 1–6 cells; mRNA level of OXPHOS-related genes was assessed by qRT-PCR (*n* = 6 per group, repeated three times). Data are presented as mean ± standard deviation (SD) from three independent biological replicates. Statistical significance was assessed using two-tailed unpaired multiple *t*-tests, with false discovery rate (FDR) correction applied to control for Type I errors. Corresponding *q*-values are indicated above each pair of bars. *q* < 0.05 was considered statistically significant. **F** Analysis of 24-h glucose consumption in Hepa 1–6 and HepG2, in which NSD2 was knocked out or overexpressed (*n* = 5 for each group; repeated three times). **G** Analysis of oxygen consumption in Hepa 1–6 and HepG2, in which NSD2 was knocked out or overexpressed (*n* = 3 for each group; repeated three times). **H** Analysis of glycolysis in Hepa 1–6 and HepG2 in which NSD2 was knocked out or overexpressed (*n* = 3 for each group; repeated three times). **I** Analysis of ATP production in Hepa 1–6, in which NSD2 was knocked out or overexpressed (*n* = 4 for each group; repeated three times).

The results above prompted us to investigate the underlying mechanisms by which NSD2 inhibits HCC. Therefore, we performed RNA-Seq analysis with liver tissues from control and NSD2^LVOE/+^ mice treated with DEN. The results of the global transcriptome were dramatically changed in NSD2^LVOE/+^ liver compared to the WT liver (Fig. 2A). Among a total of 15944 genes expressed, 1198 genes were up-regulated, and 1267 genes were down-regulated in NSD2^LVOE/+^ liver.

KEGG term analysis indicated that there was a significant enrichment of genes linked to OXPHOS, NF-kappa B signaling pathway and Non-alcoholic fatty liver disease (Fig. 2B). Interestingly, the gene set enrichment analysis (GSEA) analysis showed that NSD2^LVOE/+^ liver had lower expression of the genes related to OXPHOS (Fig. 2C). Next, RT-qPCR was performed to validate these results (Fig. 2D).

To further solidify this, the NSD2 knockout and overexpression cell lines were generated respectively from Hepa 1-6 and HepG2 (Supplementary Fig. 2A). The RT-qPCR results proved that NSD2 overexpression decreased the levels of OXPHOS-related genes, and the NSD2 deficiency increased the levels of those genes (Fig. 2E). In order to explore the changes in metabolism, we measured the glucose and lactic acid content of the cells. Indeed, glucose consumption was decreased in NSD2 overexpression cells, which was increased in NSD2 knockout cells (Fig. 2F). Meanwhile, the lactate accumulation showed no change in both NSD2 knockout and overexpression cell lines (Supplementary Fig. 2B). Consistently, we found that oxygen consumption was decreased in the NSD2 overexpression cells compared to controls (Fig. 2G). Moreover, NSD2 deficiency increases the oxygen consumption level (Fig. 2G). While the level of glycolysis showed no change in both NSD2 knockout and overexpression cell lines (Fig. 2H), this indicated that glycolysis was not affected by NSD2. Finally, NSD2 overexpression leads to reduced ATP production and decreased mitochondrial membrane potential, and NSD2 deficiency increases ATP production and mitochondrial membrane potential in Hepa 1–6 (Fig. 2I and Supplementary Fig. 2C). These results showed that NSD2 inhibits OXPHOS and decreases the glucose consumption in HCC cells.

### NSD2 inhibits OXPHOS by promoting the expression of Camk2d and Prkce

**Fig. 3:**
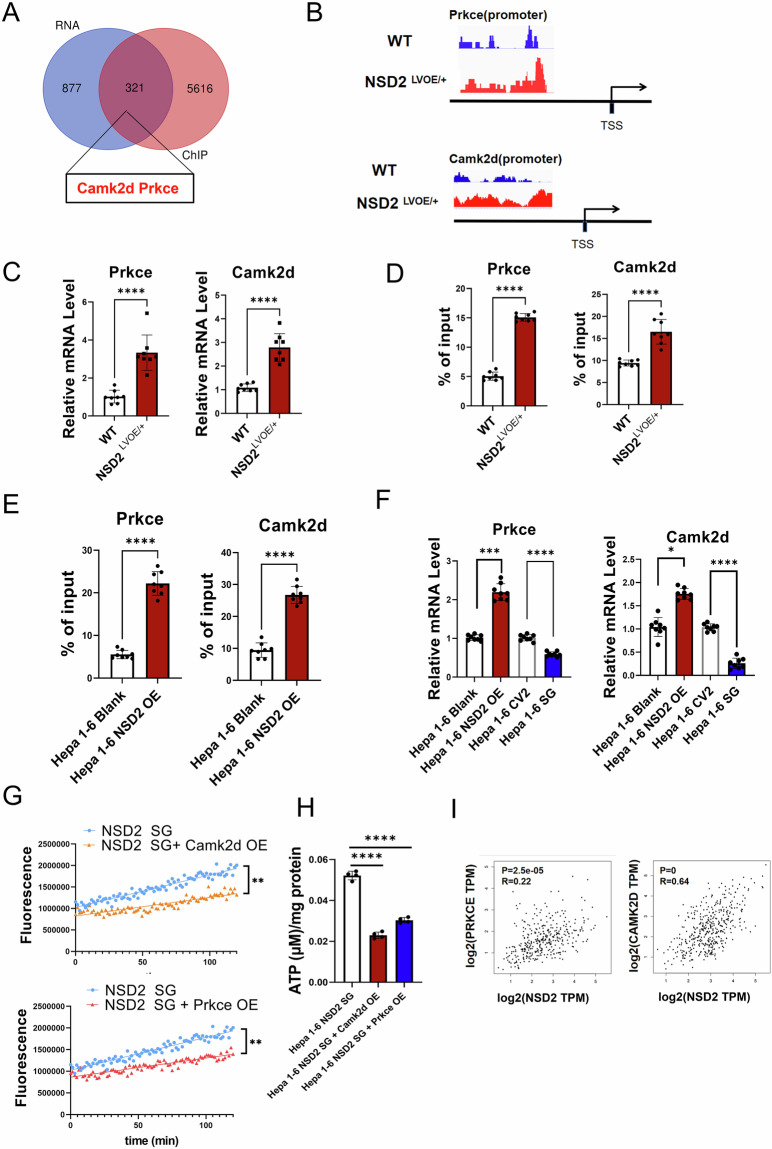
NSD2 inhibits OXPHOS by promoting the expression of Camk2d and Prkce. **A** Venn diagram showing the number of genes harboring H3K36me2 binding and displaying expression UP in NSD2LVOE/+ livers. **B** Snapshot of H3K36me2 ChIP-Seq signals at the Prkce and Camk2d gene promoter in liver from WT and NSD2LVOE/+ mice of 2-week-old mice with DEN treatment. **C** Relative mRNA expression levels of Prkce and Camk2d in liver from DEN-treated[2w] mice as indicated (*n* = 8 per group). **D** ChIP-qPCR analysis of H3K36me2 binding for Prkce and Camk2d loci in liver from WT and NSD2LVOE/+ mice of 2-week-old mice with DEN treatment (*n* = 8 per group). **E** ChIP-qPCR analysis of H3K36me2 binding for Prkce and Camk2d loci in Hepa 1–6 cells in which NSD2 was overexpressed (*n* = 88 per group). **F** Relative mRNA expression levels of Prkce and Camk2d in Hepa 1–6 in which NSD2 was knocked or overexpressed (*n* = 8 per group). **G** Analysis of Oxygen consumption in the Hepa 1–6 NSD2 SG cells in which Prkce or Camk2d was overexpressed (*n* = 3 for each group; repeated three times). **H** Analysis of ATP production in the Hepa 1–6 NSD2 SG cells in which Prkce or Camk2d was overexpressed (*n* = 4 for each group; repeated three times). **I** Correlation between NSD2 with Prkce and Camk2d expression levels in (using data from TCGA) in GEPIA. Statistical significance was determined using the Pearson correlation coefficient.

NSD2-mediated H3K36me2 in the promoter is associated with transcriptional activation. To explore the mechanistic relationship between NSD2 and OXPHOS, we conducted H3K36me2 ChIP-Seq in the hepatocytes from WT and NSD2^LVOE/+^ mice treated with DEN. The results revealed that 2734 peaks were lost and 10745 peaks were gained after NSD2 overexpression. To correlate the chromatin binding with the transcriptional regulation, we integrated the ChIP-seq data with the RNA-seq data. The Venn diagrams indicated that 321 genes showed direct H3K36me2 occupancies and upregulated expressions upon the NSD2 overexpression (Fig. 3A). As expected, Camk2d and Prkce, OXPHOs-inhibiting genes [25, 26], were listed among the 321 genes described above (Fig. 3A), and their upregulation was validated in liver cells collected from WT and NSD2^LVOE/+^ mice (Fig. 3C). The increased H3K36me2 modifications in Camk2d and Prkce were exemplified by browser tracts (Fig. 3B). We further validated the existence of H3K36me2 at the gene promoters of Camk2d and Prkce by the ChIP-qPCR assays, and found that the abundance of H3K36me2 increased along with the overexpression of NSD2 (Fig. 3D).

Next, we repeated ChIP-qPCR assays in NSD2 overexpression cell Hepa 1–6. The results also showed that the level of H3K36me2 at the promoter of Camk2d and Prkce increased after NSD2 overexpression (Fig. 3E). What’s more, Q-PCR also proved that NSD2 overexpression increased the expression levels of Camk2d and Prkce, while the NSD2 deficiency decreased the expression levels of those genes (Fig. 3F). Next, the Camk2d and Prkce overexpression cell lines were generated from NSD2 KO cells, respectively (Supplementary Fig. 3A, B). NSD2-knockout Hepa 1–6 showed a decrease in oxygen consumption level, ATP production and mitochondrial membrane potential after Camk2d and Prkce overexpression, respectively (Fig. 3G, H). Finally, we used the TCGA database to investigate their clinical relevance. Consistent with our findings, there were positive correlations between the mRNA levels of NSD2 and those of CAMK2D and PRKCE, respectively, based on the clinical database (Fig. 3I). Altogether, our results showed that NSD2 inhibited OXPHOS by promoting the expression of Camk2d and Prkce.

### Loss of NSD2 promotes the HCC progression through upregulating the expression of PD-L1

**Fig. 4:**
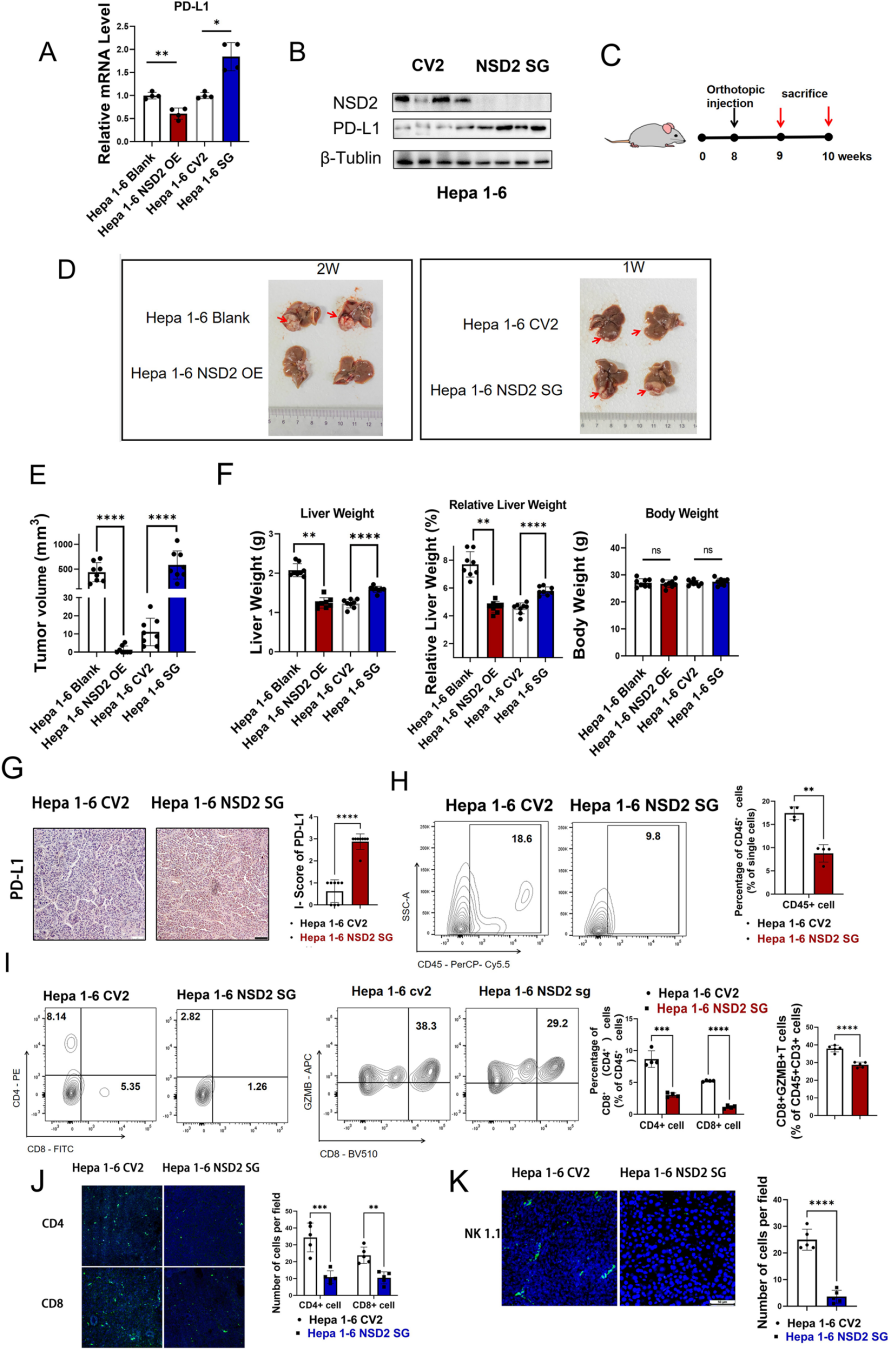
Loss of NSD2 promotes HCC progression by promoting the expression of PD-L1. **A** Relative mRNA expression levels of PD-L1 of Hepa 1–6 cells in which NSD2 was knocked out or overexpressed (*n* = 4, per group). **B** Immunoblot analyses of PD-L1 expression of Hepa 1–6 cells in which NSD2 was knocked out (repeated more than three times). **C** Schematic representation of the orthotopic injection protocol used to induce HCC in WT mice. **D** Representative image of liver accepted orthotopic injection of Hepa 1–6 cells in which NSD2 was knocked out or overexpressed (scale bars: 1 cm). **E** Tumor volume of liver accepted orthotopic injection of Hepa 1–6 cells (*n* = 8, per group). **F** Body weights, liver weights and relative liver weights of liver accepted orthotopic injection of Hepa 1–6 cells in which NSD2 was knocked out or overexpressed (*n* = 8, per group). **G** Immunohistochemical analyses of PD-L1 in liver accepted orthotopic injection of Hepa 1–6 cells in which NSD2 was knocked out or overexpressed (scale bars: 50 μm). **H** Representative flow cytometry plots and quantification of %CD45+ cells in Hepa 1–6-injected tumor (*n* = 4, per group). **I** Representative flow cytometry plots and quantification of %CD4+, %CD8+, and CD8+ GZMB+ cells in Hepa 1–6-injected tumor (*n* = 4–5, per group). **J** Immunofluorescence images and quantification of CD8+ and CD4+ cells in Hepa 1–6-injected tumor (*n* = 5, per group). **K** Immunofluorescence images and quantification of NK 1.1+ cells in Hepa 1–6-injected tumor (*n* = 5, per group).

It has been reported that OXPHOS commitment allows cells to highly express PD-L1 [22, 27, 28]. Therefore, we explored whether the overexpression of NSD2 could downregulate the expression of PD-L1 and inhibit the occurrence of HCC. RT-qPCR assays showed that PD-L1 mRNA levels were significantly decreased in the NSD2-OE Hepa 1-6 compared with those in the control cell, and were significantly increased in the NSD2-SG Hepa 1-6 compared with those in the control cell (Fig. 4A). PD-L1 protein levels were also significantly increased in the NSD2 KO cell (Fig. 4B).

It’s well known that PD-L1 expressed on the surface of cancer cells can make tumor cells escape from the recognition and killing of immune cells. In order to explore whether PD-L1 was regulated by NSD2, we used NSD2 knockout and overexpression Hepa 1–6 to perform the orthotopic injection in the liver of WT mice. The liver was collected one week or two weeks after injection for subsequent analysis (Fig. 4C). The mice receiving NSD2-SG Hepa 1–6 injection developed larger liver tumor nodules than the control, and the mice receiving NSD2-OE Hepa 1–6 injection did not develop tumor nodules (Fig. 4D, E). We also observed heavier liver weight in NSD2-SG Hepa 1–6 injected mice than the control group, and lighter liver weight in mice receiving NSD2-OE Hepa 1–6 injection (Fig. 4F).

In order to further confirm the regulation of NSD2 on PD-L1, we isolated HCC tissues from the mice receiving orthotopic injection for WB and IHC of PD-L1. Similar to what was seen in Hepa 1–6, the protein level of PD-L1 was upregulated in the HCC tissue from mice receiving NSD2-SG Hepa 1–6 injection (Fig. 4G). Flow cytometry analysis showed decreased accumulation of CD45+ cells, and decreased accumulation of CD4+ and CD8+ T cells in CD45+ cells from tumor nodules of mice receiving NSD2-SG Hepa 1–6 injection (Fig. 4H, I). Furthermore, intratumoral granzyme B-positive CD8+ (GZMB+ CD8+) T cells were also significantly decreased in tumor nodules of mice receiving NSD2-SG Hepa 1–6 injection (Fig. 4I). Finally, Immunofluorescence also showed that the number of NK cells, CD4+ T and CD8+ T cells was decreased in mice receiving NSD2-SG Hepa 1-6 injection (Fig. 4J, K). These data indicated that loss of NSD2 promotes the HCC progression through upregulating the expression of PD-L1.

### NSD2 inhibits the expression of PD-L1 by downregulating OXPHOS

**Fig. 5:**
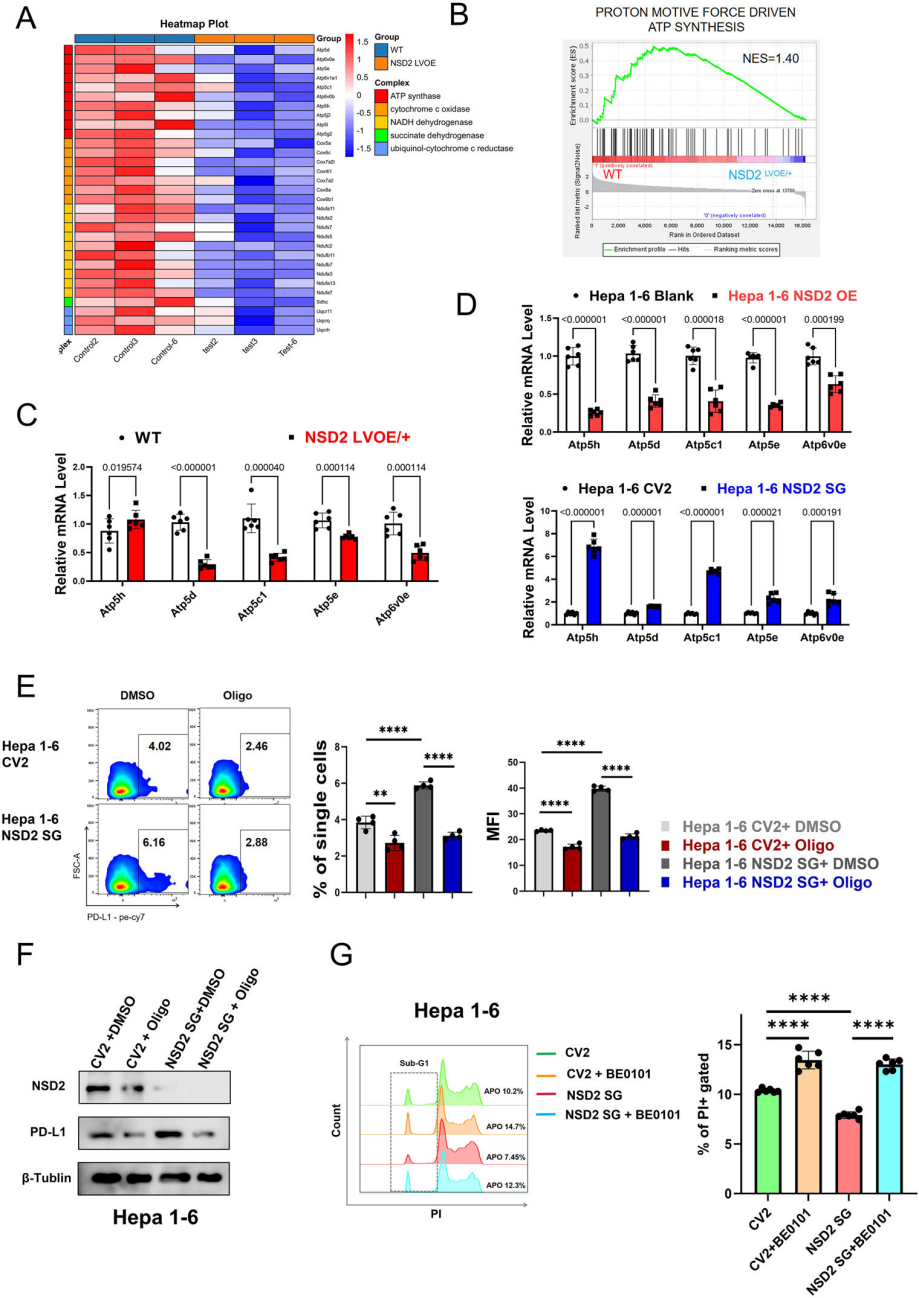
NSD2 inhibits the expression of PD-L1 by downregulating OXPHOS. **A** Heat map of RNA-seq data to compare the OXPHOS-related genes in the liver from WT and NSD2LVOE/+ mice of 2-week-old mice with DEN treatment (*n* = 3). **B** GSEA plot of enrichment in “ATP-SYNTHESIS” in WT and NSD2LVOE/+ liver. **C** Relative mRNA expression levels of complex Ⅴ-related genes in liver from DEN-treated[2w] mice as indicated (*n* = 6 per genotype). Data are presented as mean ± standard deviation (SD) from three independent biological replicates. Statistical significance was assessed using two-tailed unpaired multiple *t*-tests, with false discovery rate (FDR) correction applied to control for Type I errors. Corresponding *q*-values are indicated above each pair of bars. *q* < 0.05 was considered statistically significant. **D** Relative mRNA expression levels of complex Ⅴ-related genes of Hepa 1–6 cells in which NSD2 was knocked or overexpressed (*n* = 6 per group, repeated three times). Data are presented as mean ± standard deviation (SD) from three independent biological replicates. Statistical significance was assessed using two-tailed unpaired multiple *t*-tests, with false discovery rate (FDR) correction applied to control for Type I errors. Corresponding *q*-values are indicated above each pair of bars. *q* < 0.05 was considered statistically significant. **E** Flow cytometry analysis and quantification of PD-L1 expression in NSD2 knocked Hepa 1–6 cells in the presence or absence of oligomycin (*n* = 4 for each group, repeated more than three times). **F** PD-L1 expression was determined by Immunoblot in NSD2-knockdown Hepa 1–6 cells in the presence or absence of oligomycin (repeated more than three times). **G** Apoptosis was determined in NSD2 knocked Hepa 1–6 cells in the presence or absence of BE0101 (*n* = 6 for each group; repeated more than three times).

Mitochondrial complex V has been reported to be involved in maintaining the PD-L1 expression [22]. In order to explore the mechanism of NSD2-mediated OXPHOS regulating PD-L1, we analyzed the expression of five mitochondrial complex components in RNA-seq data. The heat map showed that the genes related to mitochondrial complex V were significantly down-regulated (Fig. 5A). Considering that mitochondrial complex V is mainly involved in ATP synthesis, we performed gene-set enrichment analysis. The results showed that the NSD2^LVOE/+^ liver had lower expression of genes related to ATP synthesis (Fig. 5B). Subsequently, we evaluated the expression of mitochondrial complex V in the liver of HCC mice. The results of Q-PCR proved that Nsd2 overexpression decreased the expression levels of Mitochondrial complex V (Fig. 5C). To further solidify our findings, the mRNA levels of mitochondrial complex V in NSD2 overexpression and knockout cell lines were also detected. Similar to what was seen in mice, mitochondrial complex V was downregulated in the NSD2-OE Hepa 1–6, and upregulated in the NSD2-SG Hepa 1–6 (Fig. 5D). To investigate the roles of mitochondrial complex V in regulating the expression of PD-L1, we used oligomycin (Oligo) to specifically block mitochondrial complex V activities and found that PD-L1 expression of NSD2-SG Hepa 1–6 reverted to control levels (Fig. 5E, F). Next, we used immune cells from the spleen of mice for co-culture experiments and found that NSD2-SG Hepa 1–6 had reduced apoptosis under co-culture conditions. After inhibiting PD-L1 by BE0101, the apoptosis of NSD2-SG Hepa 1–6 increased under co-culture conditions (Fig. 5G). Finally, RT-qPCR assays showed that PD-L1 mRNA levels were significantly increased in the NSD2-SG Hepa 1–6. What’s more, Camk2d or Prkce overexpression significantly decreased PD-L1 mRNA levels in NSD2-knockout Hepa 1–6, respectively (Supplementary Fig. 3D). Altogether, our results showed that NSD2 inhibits the expression of PD-L1 by downregulating OXPHOS.

### Either OXPHOS blocking or PD-L1 restriction is protective against NSD2-deficient HCC

**Fig. 6:**
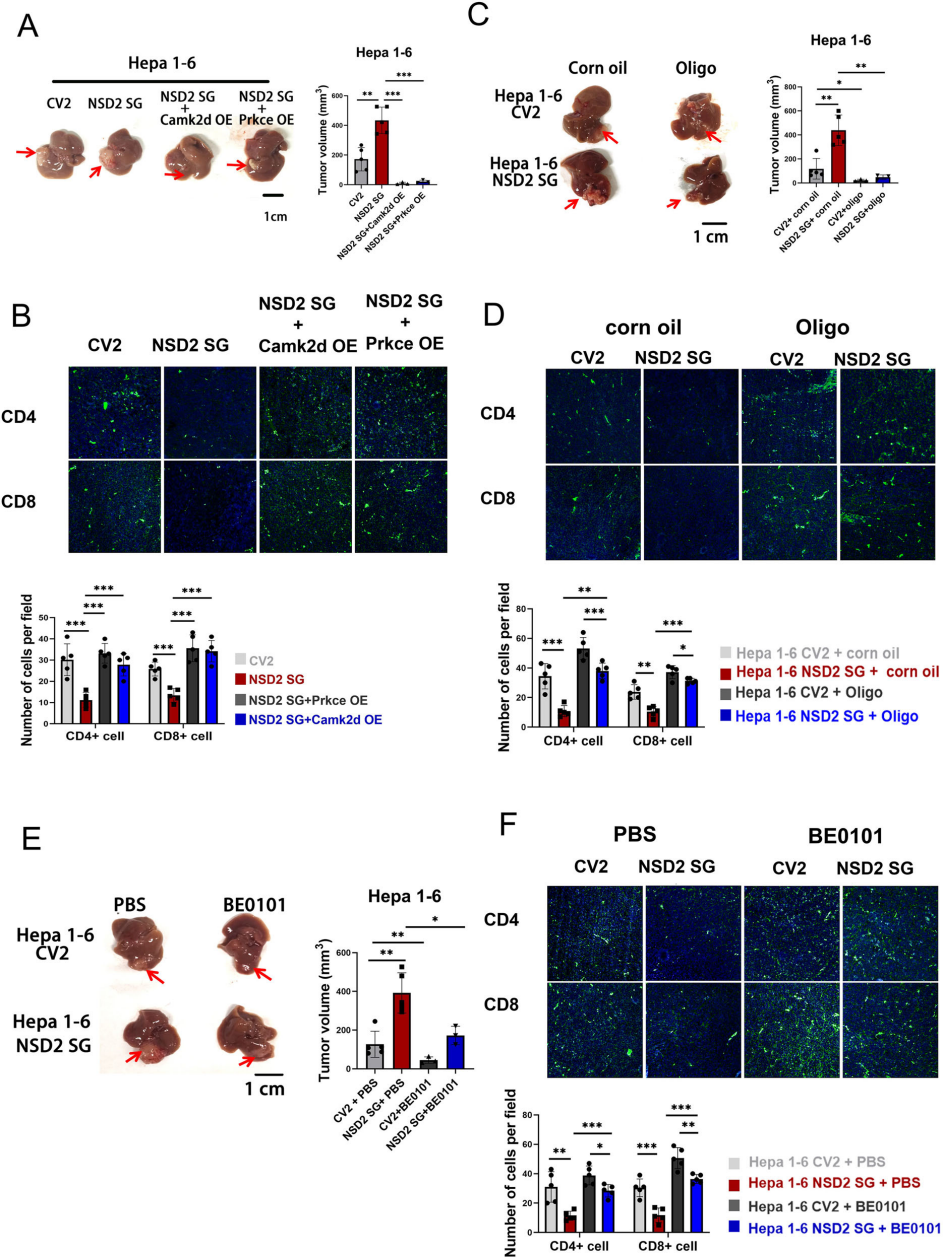
Either PD-L1 blocking or OXPHOS restriction is protective against NSD2-deficient HCC. **A** Representative image and tumor volume of liver accepted Hepa 1–6 cells injection (*n* = 3 to 5, per group) (scale bars: 1 cm). **B** Immunofluorescence images and quantification of %CD4+ and %CD8+ cells in Hepa 1–6-injected tumor (*n* = 5, per group). **C** Representative image and tumor volume of liver accepted Hepa 1–6 SG cells injection in the presence or absence of oligo (*n* = 3 to 5, per group) (scale bars: 1 cm). **D** Immunofluorescence images and quantification of %CD4+ and %CD8+ cells in Hepa 1–6 SG-injected tumor in the presence or absence of oligo (*n* = 5, per group). **E** Representative image and tumor volume of liver after Hepa 1–6 SG cells injection in the presence or absence of BE0101 (*n* = 3 to 5, per group) (scale bars: 1 cm). **F** Immunofluorescence images and quantification of %CD4+ and %CD8+ cells in Hepa 1–6 SG-injected tumor in the presence or absence of BE0101 (*n* = 5, per group).

Next, we explored whether blockade of PD-L1 or OXPHOS could alleviate tumor growth in the NSD2-deficient HCC. We performed the orthotopic injection with Camk2d and Prkce overexpression NSD2-SG Hepa 1–6. The mice receiving Camk2d and Prkce overexpression NSD2-SG Hepa 1–6 injection developed small liver tumor nodules respectively than control (Fig. 6A). The accumulation of CD45+ cells, CD4+ and CD8+ T cells were increased in tumor from mice receiving Camk2d and Prkce overexpression NSD2-SG Hepa 1–6 injection than control (Fig. 6B and Supplementary Fig. 4A). Next, we used oligomycin and BE0101 to perform rescue experiments on mice receiving orthotopic injection. Oligo treatment alleviated tumor growth in mice receiving Hepa 1–6 injection. Meanwhile, mice receiving NSD2-SG Hepa 1–6 injection generated small liver tumor nodules than controls after oligo treatment (Fig. 6C). Immunofluorescence and flow cytometry analysis showed increased accumulation of CD45+ cells, and increased accumulation of CD4+, CD8+ T and GZMB+ CD8+ T cells in tumor nodes from oligo-treated mice (Fig. 6D and Supplementary Fig. 4B). The similar results were also observed in BE0101-treated mice (Fig. 6E, F and Supplementary Fig. 4C). Altogether, these results demonstrated that both PD-L1 and OXPHOS restriction are protective against NSD2-deficient HCC.

### NSD2 inhibits the progression of HCC through the OXPHOS/PD-L1 axis

**Fig. 7:**
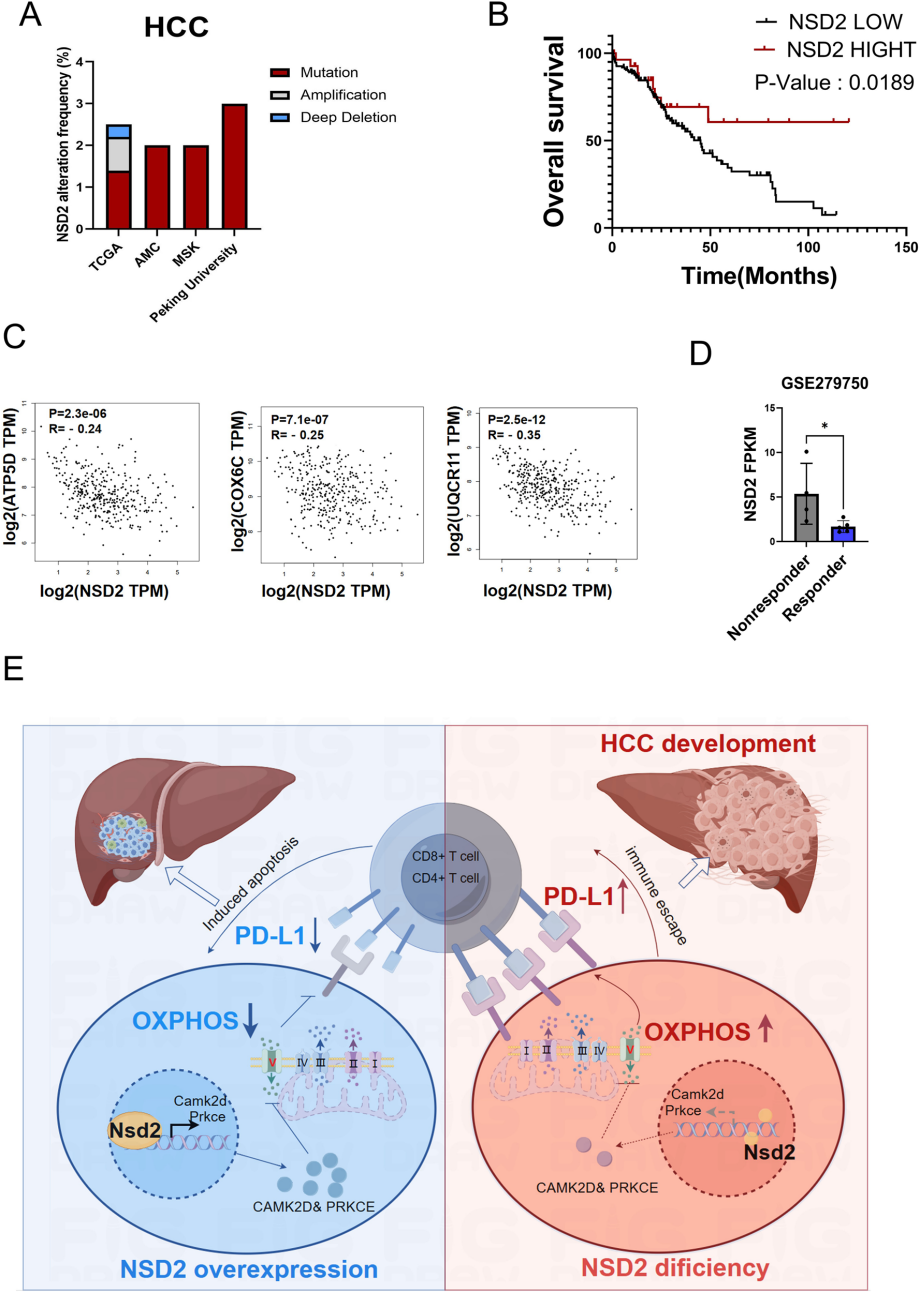
NSD2 inhibits the progression of HCC through the OXPHOS/PD-L1 axis. **A** The distribution of human NSD2 mutation in HCC patients from the different databases. **B** Overall survival of HCC patients with or without NSD2 high expression (HR = 0.3575, *p* = 0.0189). Cox proportional hazards regression analysis was employed to adjust for confounding factors. **C** Correlation between NSD2 and ATP5D, COX6C and UDCR11 expression levels in (using data from TCGA) in GEPIA. Statistical significance was determined using the Pearson correlation coefficient. **D** Quantification of NSD2 expression in HCC patients with PD-L1 inhibitor treatment (*n* = 4–5, per group). **E** Schematic presentation of the role of NSD2 in the formation of HCC.

To confirm the role of NSD2 in HCC patients, we surveyed different cancer-type databases and found that NSD2 has a mutation rate of 2–3% across different databases (Fig. 7A). Furthermore, patients with low expression of NSD2 exhibited a poor clinical survival (Fig. 7B), which suggested a suppressive role of NSD2 in HCC. Considering that NSD2 inhibits the progression of HCC by inhibiting the expression of PD-L1 through OXPHOS, we surveyed clinical database of HCC and the results showed that NSD2 was negatively correlated with OXPHOS genes (ATP5D, COX6C and UQCR11), respectively (Fig. 7C). What is more, we analyzed the RNA-sequencing data of clinical patients receiving PD-L1 treatment (GSE279750), and the results showed that patients with low expression of NSD2 have a better response to PD-L1 inhibitor treatment (Fig. 7D). Altogether, these results showed that NSD2 inhibits the progression of HCC through OXPHOS/PD-L1 axis (Fig. 7E).

## Discussion

In this study, we discovered that patients with low expression of NSD2 experienced a poor clinical survival period. During the development of DEN-induced HCC in immunocompetent mice, the number and size of liver tumors were significantly reduced, and the liver injury markers in serum were also decreased. Subsequently, we found that NSD2 overexpression inhibited the OXPHOS pathway through promoting the target genes (Camk2d and Prkce) transcription by modulating the activity of promoters. The down-regulation of the OXPHOS pathway leads to the suppression of PD-L1 expression on the surface of tumor cells, so that tumor cells can be better recognized and killed by immune cells. Altogether, our study demonstrates that NSD2 acts as a tumor suppressive factor during the formation of HCC.

As a histone methyltransferase, NSD2 participates in the occurrence and development of various solid tumors [29, 30]. Abhijit et al. demonstrated that NSD2 acts as an essential subunit of the AR/FOXA1 neo-enhanceosome to drive prostate tumorigenesis [31]. Da et al. revealed that NSD2 promotes tumor angiogenesis via methylating and activating STAT3 [32]. Danyang et al. showed that NSD2 promotes TNBC progression via the NSD2-H3K36me2-ULK1-autophagy axis [33]. Previous studies have primarily focused on the roles of NSD2 in regulating tumor cell proliferation and invasion. Here, we found that NSD2 enhances the anti-tumor immunity to inhibit the formation of HCC through the OXPHOS/PD-L1 axis in HCC. Recent studies have also revealed that the OXPHOS pathway is up-regulated in HCC [34], indicating that OXPHOS is the main driving pathological feature of HCC. In our study, we found that inhibition of OXPHOS mediated by NSD2 overexpression can inhibit the formation of HCC, while loss of NSD2 promotes the formation of HCC. Moreover, it has been reported that OXPHOS can promote the expression of PD-L1 in macrophages [22]. Although this article reports that mitochondrial complex V can modulate PD-L1 expression, and our results also showed that overexpression of NSD2 inhibits the function of mitochondrial complex V, the specific mechanisms by which oxidative phosphorylation regulates PD-L1 remain to be further elucidated. And our results showed that inhibition of OXPHOS in HCC down-regulates the expression of PD-L1 to regulate anti-tumor immunity.

It is worth pointing out that previous research reported that NSD2 facilitates the proliferation, migration and invasion of HCC cell lines [24]. However, our results demonstrate that overexpression of NSD2 inhibits the occurrence and development of HCC by regulating PD-L1 in primary HCC models. Meanwhile, the proliferation capacity of NSD2-knockout HCC cell lines is enhanced in orthotopic injection models. The regulation of the tumor immune microenvironment plays a pivotal role in tumor development, which may lead to inconsistent function of genes between the vitro and vivo [35, 36]. Considering the tumor microenvironment and liver metabolites in HCC, our HCC models may be closer to the physiological status of HCC. Therefore, NSD2 suppresses the formation and progression of HCC in vivo.

In this study, we focused on hepatocellular carcinoma patients who received PD-L1 inhibitor treatment and had available information on treatment response, which led to the relatively small sample size. Despite this constraint, statistical analysis confirmed significant differences in NSD2 expression between responders and non-responders, supporting the clinical relevance of our findings. We will continue to expand the clinical validation by integrating more eligible GEO datasets or prospective clinical samples in future studies.

In the past years, NSD2 has been considered a cancer-promoting factor. However, with the deepening of research on NSD2, the anti-tumor effect of NSD2 began to be discovered. Ren et al. reported that NSD2 can inhibit the development of colorectal cancer through the IFN-γ signaling pathway [37]. Notably, our data firstly reveal a tumor-suppressive function of NSD2 in HCC. The loss of NSD2 leads to increased expression of PD-L1 on the tumor cells, and the blockade of PD-L1 or OXPHOS can inhibit tumor proliferation caused by NSD2 loss. Whether the combination of PD-L1 blockade and OXPHOS inhibition has a stronger suppressive effect on HCC needs to be further studied. Combined treatment with OXPHOS inhibitors and PD-L1 blockade may yield superior therapeutic outcomes in HCC patients with immune checkpoint inhibitor resistance. This result suggests that HCC patients with NSD2 mutations may be more sensitive to PD-L1 inhibitor therapy. In summary, our study identifies the key role of NSD2 in the immune surveillance of HCC and provides new ideas for the drug development of HCC. Activation of NSD2 may represent a potential therapeutic modality for hepatocellular carcinoma.

## Supplementary information

Supplemental material

## Data Availability

The datasets used and/or analyzed during the current study are available from the corresponding author on reasonable request.
