# Supplementary material for: NSD2 inhibits the expression of PD-L1 via oxidative phosphorylation to control immune surveillance in hepatocellular carcinoma
Source: Cell Death Dis. 2026 Feb 27;17(1):284. doi: 10.1038/s41419-026-08490-x (PMC13031530; doi:10.1038/s41419-026-08490-x)

# Supplement Methods

**Chromatin immunoprecipitation assay**

For chromatin immunoprecipitation assay, 100 mg of tissue was cut into 1 mm3 pieces in PBS containing protease inhibitors, and then cross-linked with 1 % formaldehyde at room temperature for 10 minutes, and planted with 0.125 M glycine for 5 minutes. The cross-linked tissue was homogenized and centrifuged to collect the precipitate. Next, the cells were resuspended with lysis buffer (0.1 % SDS and 5 mM EDTA, 50 mM Tris-HCl [ pH 8.0 ]) and incubated at 4 °C for 5 minutes. After centrifugation, the precipitate was washed twice with cold PBS and collected in a ChIP digestion buffer. (50 mM of Tris-HCl [pH 8.0], 1 mM of CaCl2, and 0.2% Triton X-100). DNA was digested by MNase ( M0247S; nEB ) and was cut to 150-300 bp. Each sample was diluted to 1 mL with dilution buffer ( 20 mM Tris-HCl [ pH 8.0 ], 150 mM NaCl, 2 mM EDTA, 1 % Triton X-100, and 0.1 % SDS ) and incubated with protein G magnetic beads and H3K36me2 antibodies at 4 °C for 10 h. The beads were washed 5 times and the DNA was eluted with ChIP elution buffer. (0.1 M of NaHCO3, 1% SDS, 20 µg/mL of proteinase K). The eluent was incubated overnight at 65 °C, and a DNA purification kit ( DP214-03; tiangen ) was used to extract DNA. The purified DNA was subjected to qPCR or high-throughput sequencing using the Illumina Hiseq X Ten platform.

**RNA-seq and data analysis**

The NEBNext PolyA mRNA Magnetic Isolation Module (New England Biolabs, Ipswich, MA, USA) was used to performe mRNA isolation, and the mRNA was then used for RNA-Seq library preparation with the NEB Next Ultra Directional RNA Library Prep Kit for Illumina (New England Biolabs, Ipswich, MA, USA). The library was then subjected to Illumina sequencing with paired-end 2x150 as the sequencing mode. Raw reads were filtered to obtain high-quality clean reads by removing sequencing adapters, short reads (length <35 bp) and low-quality reads using Cutadapt (v1.9.1) and Trimmomatic (v0.35). Then FastQC is used to ensure high read quality. The clean reads were mapped to the mouse genome (assembly GRCm38) using the HISAT2 software. Gene expression levels were estimated using FPKM (fragments per kilobase of exon per million fragments mapped) by StringTie. Ballgown, an R package, was used to measure differential gene expression. The false discovery rate (FDR) control method was used to calculate the adjusted P-values in multiple tests to evaluate the significance of the differences. The gene annotation file was retrieved from the Ensembl genome browser 90 database (http://www.ensembl.org/index.html). To annotate genes with gene ontology (GO) terms and KEGG pathways, ClusterProfiler (R package) was used. The functional enrichment analysis (GO and KEGG) was also performed with ClusterProfiler.

# Supplement Figure legends

**Supplement Figure 1. NSD2 inhibits the formation of HCC in mice**

1. Representative images of tumor from nude mice ( n = 5 per group, Scale Bars: 1 cm).
2. Quantification of tumor weight and tumor volume from nude mice mice (n = 5 per group).
3. Representative images of liver and liver weight from WT and NSD2^LVOE/+^ mice ( Scale Bars: 1 cm).
4. Body weight and relative liver weight from WT and NSD2^LVOE/+^ mice (n = 3 per group).
5. Quantification and representative Masson staining of liver tissue from 9-month-old WT and NSD2^LVOE/+^ mice with DEN treatment (n = 10 per group).
6. Representative NK 1.1 & F 4/80 staining and quantitation of positive cells in liver tissue from 9-month-old WT and NSD2LVOE/+ mice with DEN treatment (Scale Bars: 75 μm).

**Supplement Figure 2. Lactate accumulation and glycolysis in NSD2 knockout and overexpression cell lines**

1. Relative NSD2 mRNA expression levels in NSD2 knockout and overexpression cell lines (n = 3 per group).
2. Lactate accumulation in NSD2 knockout and overexpression cell lines (n = 3 per group, experiment was repeated three times).
3. Representative JC-1 staining and quantitation in NSD2 knockout and overexpression cell lines (n = 6 per group). (Scale Bars: 50 μm).

**Supplement Figure 3. Camk2d and Prkce overexpression cell lines were generated from NSD2 KO cells**

1. Relative Prkce mRNA expression levels in NSD2 SG and NSD2 SG + Prkce OE cell lines (n = 3 per group).
2. Relative Camk2d mRNA expression levels in NSD2 SG and NSD2 SG + Camk2d OE cell lines (n = 3 per group).
3. Representative JC-1 staining and quantitation in NSD2 SG, NSD2 SG + Camk2d OE and NSD2 SG + Prkce OE cell lines (n = 6 per group). (Scale Bars: 50 μm).

**Supplement Figure 4. Flow cytometry analysis of tumor nodes from mice receiving NSD2-SG Hepa 1-6 injection**

(A) Representative flow cytometry plots and quantification of %CD45+ , %CD4+ and %CD8+ cell from Hepa 1-6 -injected tumor.

(B) Representative flow cytometry plots and quantification of %CD45+ , %CD4+ , %CD8+ and CD8+GZMB+ cell from Hepa 1-6 SG-injected tumor in the presence or absence of oligo.

(C) Representative flow cytometry plots and quantification of %CD45+ , %CD4+, %CD8+ and CD8+GZMB+ cell from Hepa 1-6 SG-injected tumor in the presence or absence of BE0101.


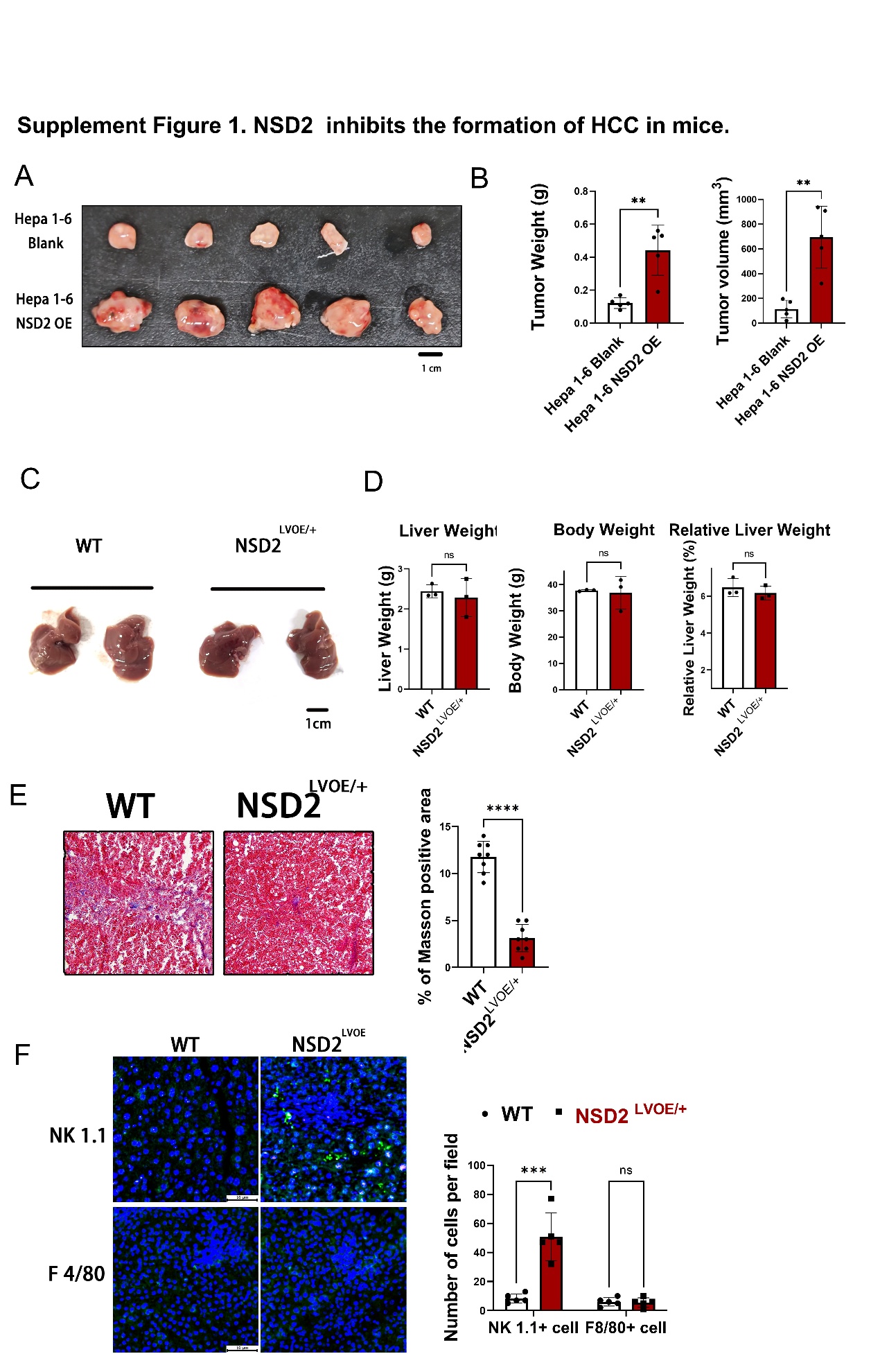

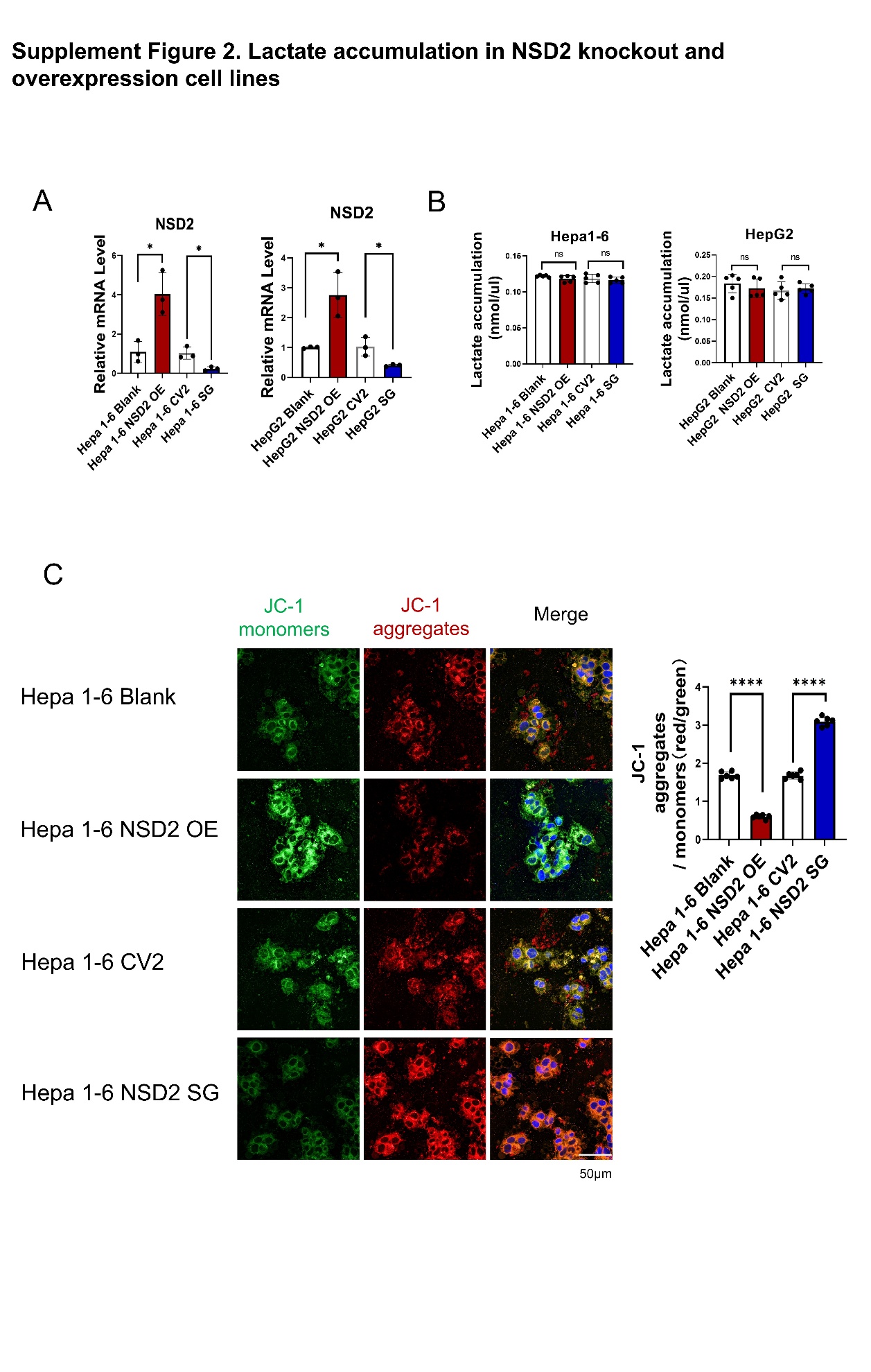

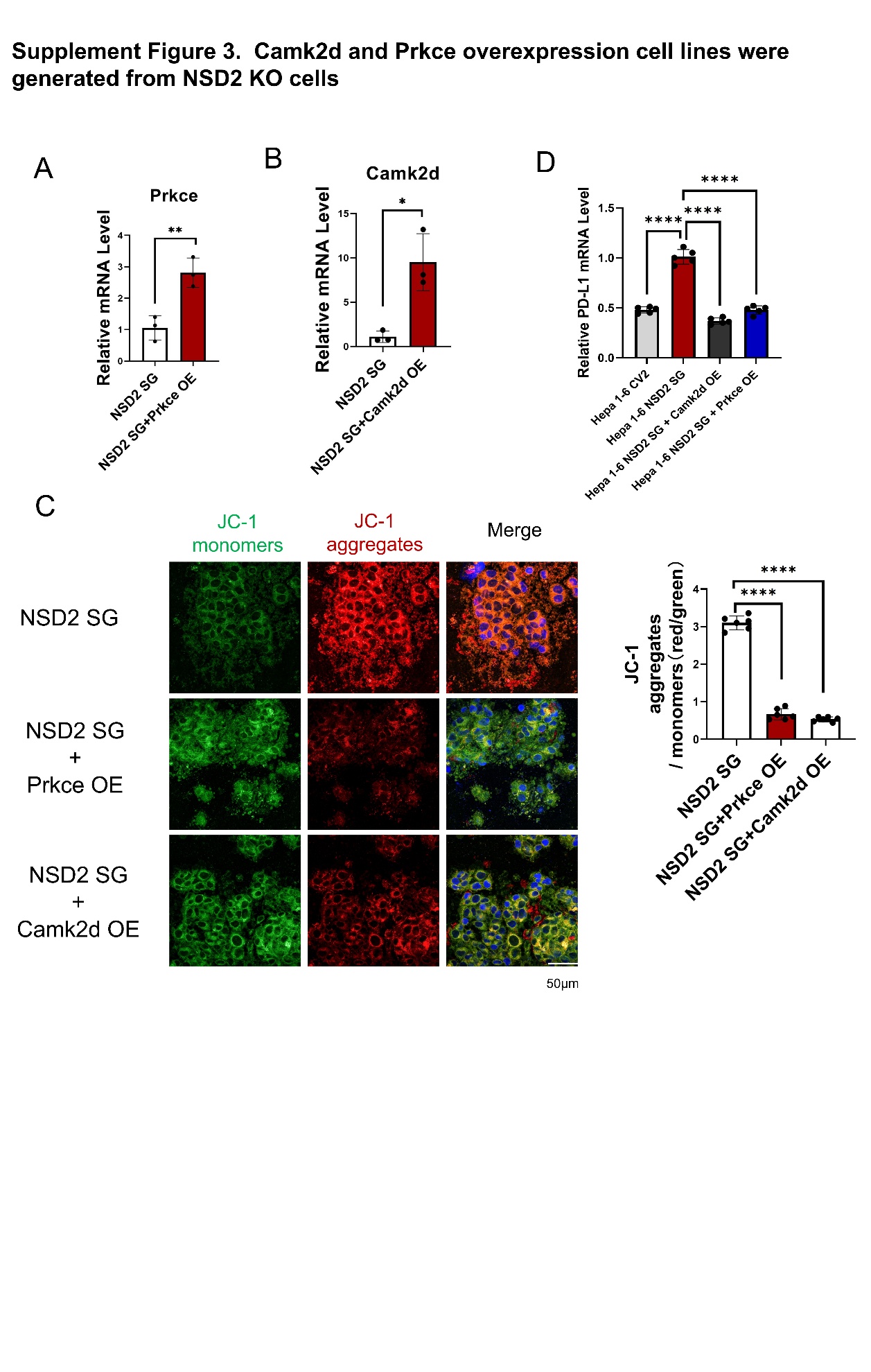

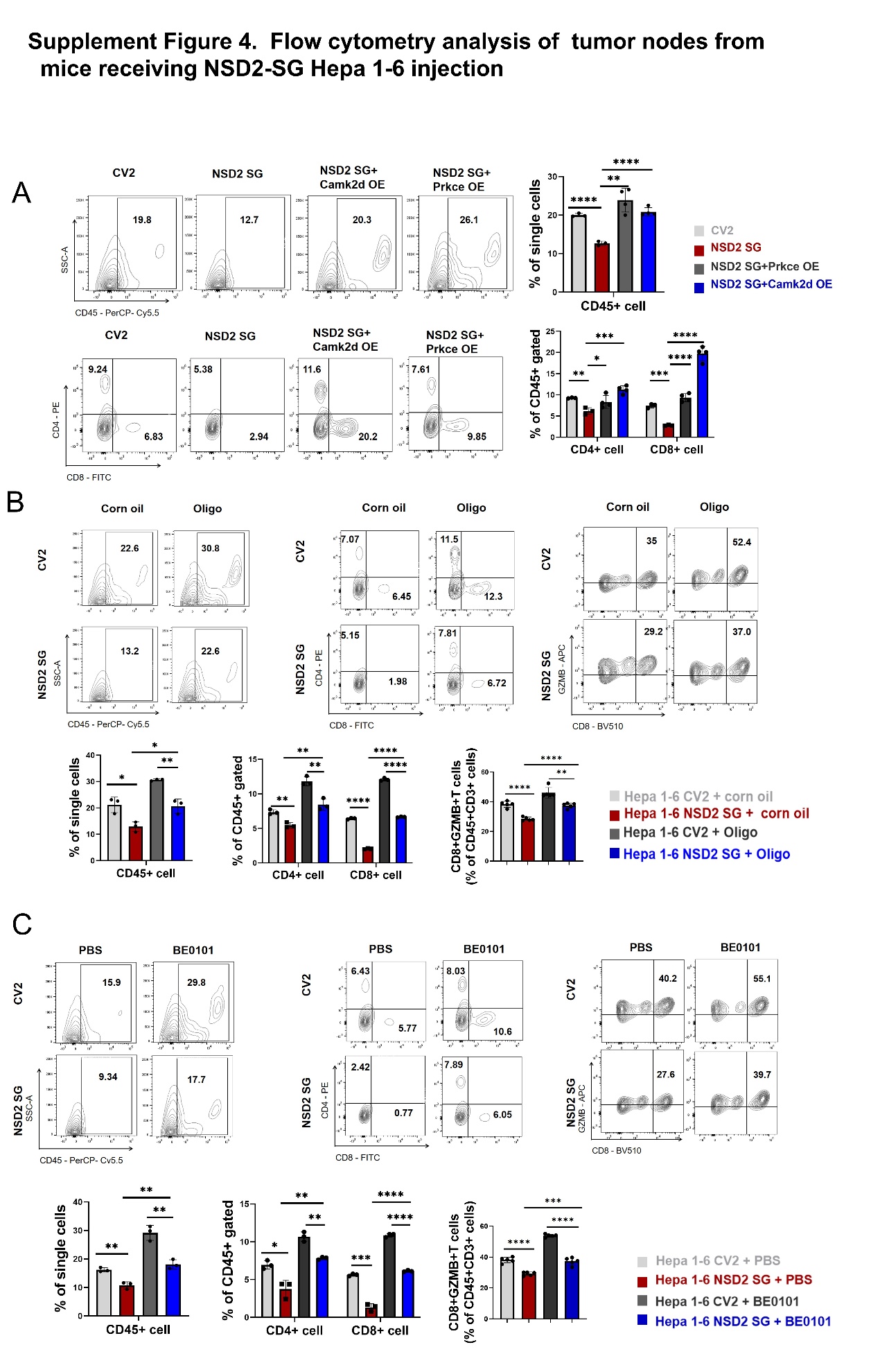

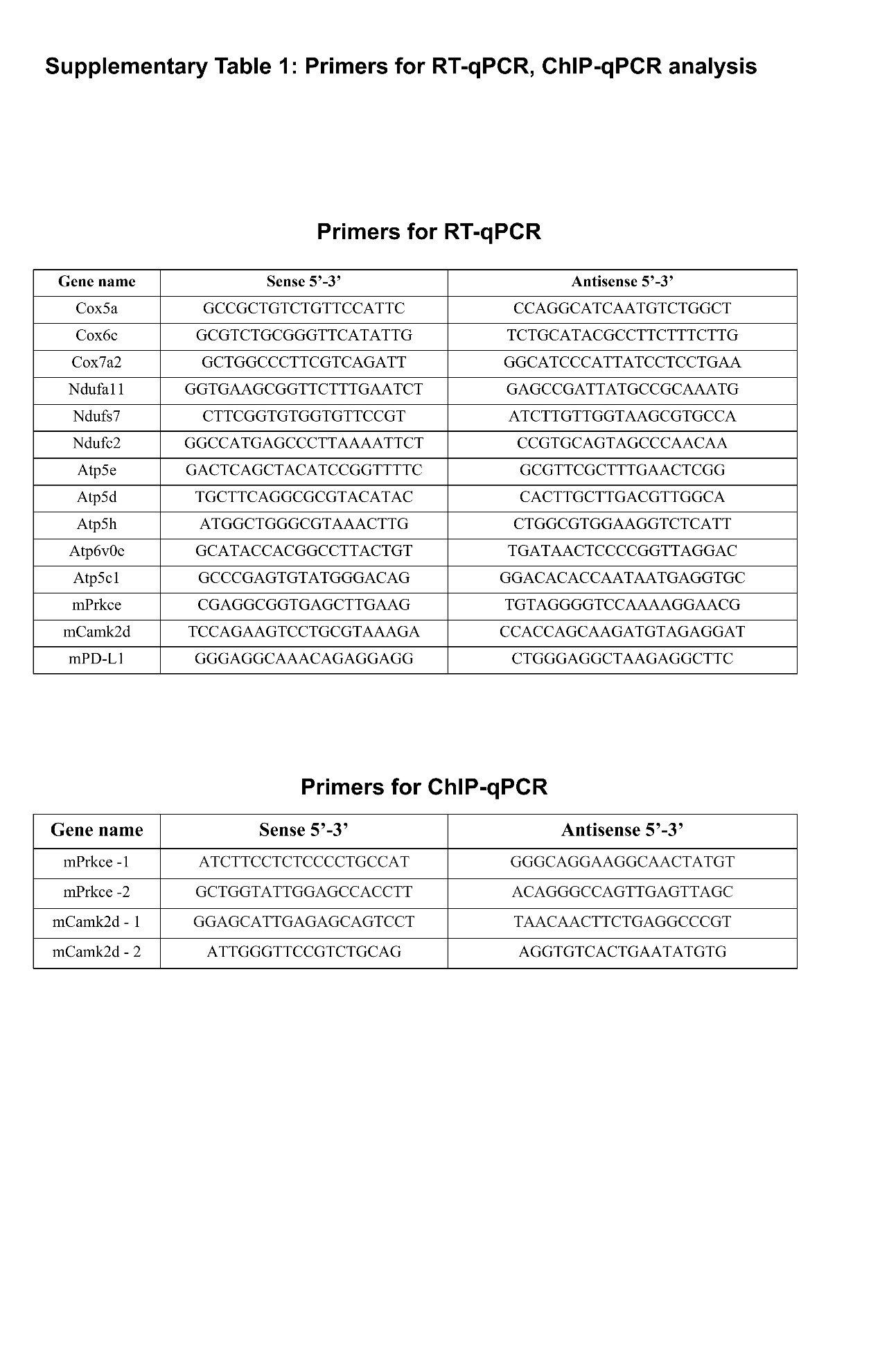

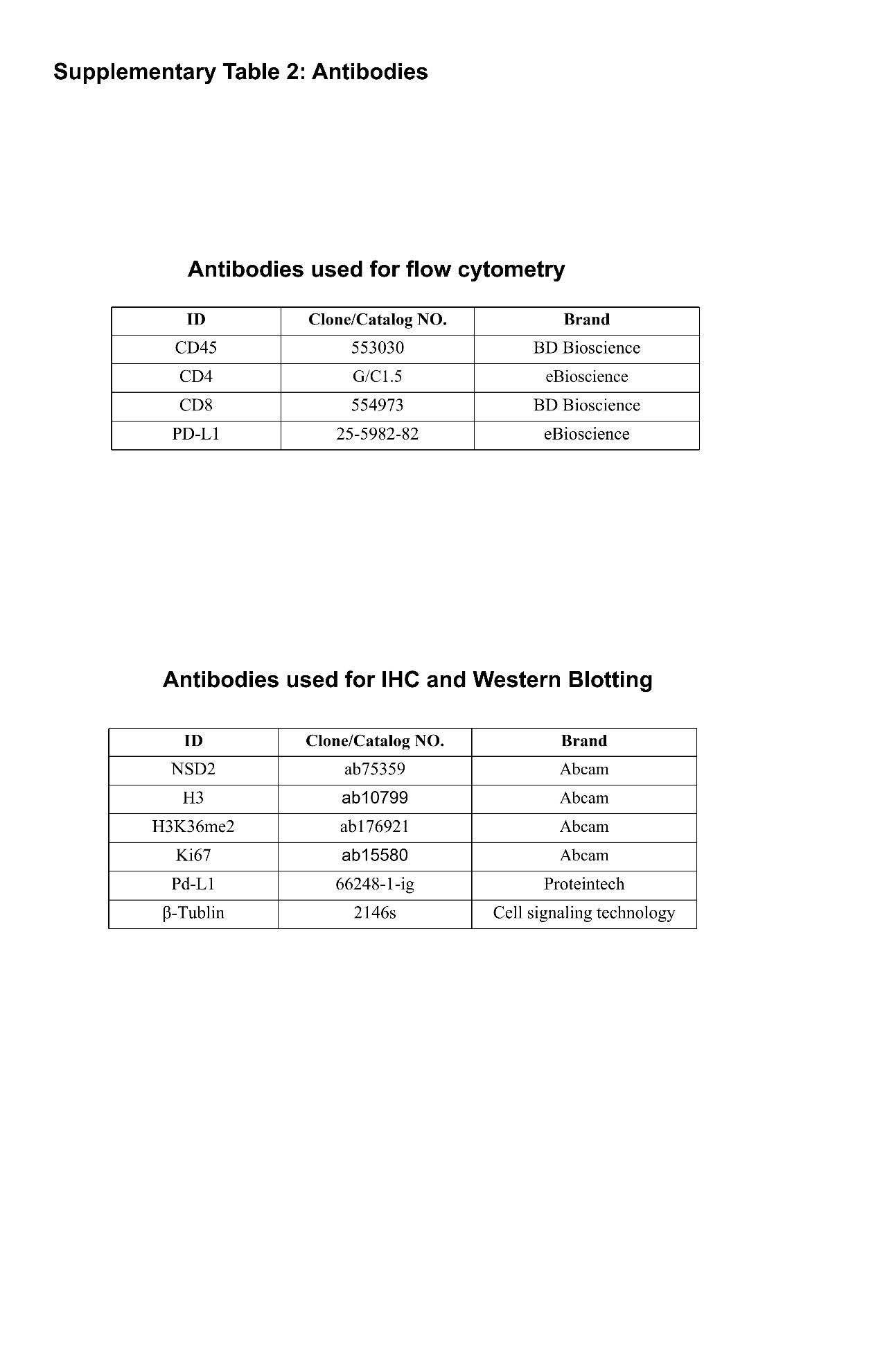

Supplement: Supplementary file 1 — Supplemental material [file 41419_2026_8490_MOESM1_ESM.docx]
